# Side-Dependent Trunk Muscle Modulation During Sit-to-Stand After Stroke: An Exploratory EMG and Kinematic Study

**DOI:** 10.3390/s26082353

**Published:** 2026-04-10

**Authors:** Grazia Cravero, Alice De Luca, Beatrice Lagomarsino, Carmelo Lentino, Giorgia Marchesi, Debora Siri, Camilla Pierella, Maura Casadio

**Affiliations:** 1 Department of Informatics, Bioengineering, Robotics and Systems Engineering, University of Genoa, 16145 Genoa, Italy; beatrice.lagomarsino@edu.unige.it (B.L.); s4362018@studenti.unige.it (D.S.); camilla.pierella@unige.it (C.P.); maura.casadio@unige.it (M.C.); 2 Italian Institute of Technology, 16145 Genoa, Italy; alice.deluca@iit.it; 3 Recovery and Functional Reeducation Unit, Rehabilitation Department, Santa Corona Hospital, 17027 Pietra Ligure, Italy; c.lentino@asl2.liguria.it; 4 Movendo Technology s.r.l., 16149 Genoa, Italy; giorgia.marchesi@movendo.technology

**Keywords:** sit-to-stand, stroke, trunk muscles, electromyography, kinematics

## Abstract

Sit-to-stand (STS) is a fundamental functional task frequently impaired after stroke and widely used in rehabilitation to assess motor control and balance. While lower-limb kinematic and muscular asymmetries during STS have been documented, the contribution of trunk muscle coordination to compensatory strategies has received limited attention. We investigated STS performance in seven individuals with chronic right-sided hemiparesis under two conditions (free arms and crossed arms) to characterize phase-dependent kinematic asymmetries and side-dependent trunk muscle modulation relevant to rehabilitation practice. Optoelectronic motion capture was synchronized with bilateral surface electromyography, providing time-aligned kinematic and neuromuscular signals for sensor-based assessment of STS. Participants exhibited prolonged and highly variable STS durations, along with ankle asymmetries during the rising and lowering phases and hip asymmetries during upright standing, indicating increased reliance on the less impaired limb. Electromyography revealed side-dependent modulation of trunk muscles, notably latissimus dorsi, erector spinae longissimus, and multifidus, characterized by a prolonged relative contribution on the more impaired side. These findings suggest that altered trunk muscle modulation contributes to compensatory STS strategies after stroke and highlight the importance of trunk-focused neuromuscular assessment to guide individualized rehabilitation interventions aimed at improving symmetry, postural stability, and movement efficiency.

## 1. Introduction

After a stroke, subjects typically experience a range of impairments such as muscle weakness, spasticity, sensory deficits, and balance dysfunction that reduce independence, impacting essential activities of daily living [1]. Preserving autonomy after stroke is essential for quality of life, and functional abilities such as independent standing and walking are key determinants of this outcome [1,2,3]. The sit-to-stand (STS) test is widely used in clinical settings to evaluate motor control and stability in stroke subjects with functional limitations, as it requires coordinated interaction of linked body segments, effective balance control, and force distribution [4,5]. The biomechanic of STS in stroke subjects is influenced not only by stroke-induced impairments, such as paresis and loss of selective movement control, but also by compensatory strategies that can further disrupt movement patterns [6,7]. These adaptations often result in inefficient or unstable movement, making STS particularly challenging for individuals with stroke. Most investigations in this area have described kinetic and kinematic variables, obtained using force plates or from motion capture systems [8], detailing the fundamental movement phases, which include trunk and hip flexion to shift the center of mass forward, followed by extension of the lower limbs and trunk to lift the body vertically [5]. Kinematic analyses have shown that stroke individuals require more time to complete the STS movement, and exhibit excessive forward trunk flexion and excessive tilt and rotation in the frontal and transverse planes toward the affected side [2,4]. The forward displacement of the center of gravity results in increased ankle dorsiflexion values with respect to healthy unimpaired individuals [4].

The neuromuscular control of STS after stroke has been investigated, primarily focusing on lower-limb muscle activity [1,8,9,10,11]. Compared to healthy unimpaired subjects, anticipatory postural adjustments are altered in both limbs of individuals with stroke [10]. Previous studies have reported bilateral asymmetries in muscle activation timing and amplitude, with delayed and reduced recruitment on the affected side and compensatory activation on the non-paretic side [8,9,11].

Despite the recognized importance of trunk control for postural stability and functional recovery after stroke [12,13,14], a systematic review by Antiya and Ganvir [15] highlighted that only a limited number of electromyography (EMG) studies have investigated trunk muscle involvement during STS. For instance, Lee et al. [1] reported delayed activation of deep abdominal muscles, including the transversus abdominis and internal oblique, on the affected side. Similarly, Nam et al. [16] showed increased erector spinae activation on the affected side under specific task conditions, suggesting that trunk recruitment may adapt to altered lower-limb mechanics. However, these studies remain limited in scope and do not provide a comprehensive characterization of trunk muscle coordination during STS.

Evidence from other trunk-related tasks further supports the relevance of this gap. Previous work has shown altered and side-dependent activation of trunk muscles such as the rectus abdominis, latissimus dorsi, multifidus, and erector spinae during voluntary trunk movements and control exercises in individuals with stroke [17,18]. Taken together, these findings suggest a broader reorganization of trunk neuromuscular control after stroke. However, its role during STS, a task requiring coordinated trunk and lower-limb interaction, remains insufficiently characterized. To address the limited research in this area, our exploratory study analyzed the combined biomechanics and trunk muscle activation patterns during the STS movement in adults with hemiparetic stroke, comparing the more and less impaired sides. The full STS cycle was considered, including both the sit-to-stand and the subsequent return-to-sit phase. We integrated synchronized optical motion capture and surface electromyography (sEMG) to quantify trunk and lower-limb coordination during this functional task. Surface electromyographic sensors are a widely used, non-invasive technology to assess neuromuscular activation; in trunk muscle evaluation, they enable characterization of recruitment timing and relative amplitude beyond what can be inferred from clinical observation alone. Motion capture complements sEMG by providing time-resolved joint and segment kinematics, allowing movement-phase segmentation and the quantification of side-dependent biomechanical asymmetries across the STS cycle. The combined interpretation of movement kinematics and EMG modulation allows a sensor-based characterization of compensatory strategies. Unlike previous studies that focused primarily on lower-limb muscle activity or examined trunk muscles in isolation or in non-STS tasks, this study investigates the modulation of multiple trunk muscles together with whole-body kinematics across the entire STS cycle, enabling a phase-specific analysis of side-dependent compensatory strategies. Lower-limb kinematics and gluteal muscle activity were included to contextualize trunk muscle behavior within the global biomechanical strategy of the task, allowing the identification of stabilization mechanisms that emerge when propulsion or control from the lower limbs is reduced.

By identifying key neuromuscular and biomechanical impairments affecting STS performance, this research provides quantitatively derived, sensor-based markers to support clinicians in objectively characterizing trunk-related compensatory strategies after stroke. Rather than relying solely on observational clinical scales, the proposed multimodal sensing framework enables phase-specific integration of synchronized kinematic and EMG signals, offering reproducible and instrumented indicators of trunk–lower limb coordination. This exploratory study aims to identify sensor-derived patterns that may inform future hypothesis-driven investigations in larger clinical cohorts.

## 2. Materials and Methods

### 2.1. Participants

Data were collected from seven stroke participants (5 males, 2 females), who underwent a functional evaluation protocol at the Recovery and Functional Re-education Unit of the Santa Corona Hospital in Pietra Ligure, Italy. The inclusion criteria were: (i) age between 18 and 80 years old, (ii) right hemiparesis resulting from a stroke, (iii) chronic phase of the condition (i.e., more than one year after the stroke event), iv) no other musculoskeletal or neurological disorders, (v) able to stand up and walk without a cane, (vi) mini-mental state above 24. The participants had a mean age of 68.7 ± 9.0 years (mean ± standard deviation). The study was conducted in accordance with the national guidelines and the ethical standards outlined in the Declaration of Helsinki (2013 revision). It was approved by the institutional review board of the Santa Corona hospital (55/2012/CE2). All participants signed an informed consent, which included consent for the data analysis for scientific purposes and the publication of the results.

### 2.2. Experimental Setup and Protocol

Participants were instructed to stand up from an adjustable-height backless chair at a self-selected and comfortable speed and then sit back down, repeating the task five times consecutively. The seat height was adjusted based on each participant’s anthropometric measures to ensure a standardized seated posture, with feet in contact with the floor and knees at approximately 90° of flexion at the start of the task. The STS task was performed under two conditions: with arms free, allowing participants to use their arms to assist in standing up, and with arms crossed over the chest to limit upper-limb assistance.

### 2.3. Data Recording

sEMG and kinematic data were recorded. Bipolar surface electromyographic sensors were positioned according to SENIAM guidelines to ensure reliable signal acquisition from trunk muscles. Signal processing procedures were implemented to enhance signal-to-noise ratio and ensure reproducibility of sensor-derived metrics. The kinematic data were collected using a motion capture system SMART DX (BTS Bioengineering, Milan, Italy), which consisted of 8 infrared cameras, 2 video cameras, and reflective spherical passive markers of 1.5 cm in diameter. The hardware synchronization between the motion capture system and the sEMG device (BTS Bioengineering, Milan, Italy) ensured precise temporal alignment between kinematic events and neuromuscular activation patterns, enabling reliable phase-specific sensor fusion and integrated biomechanical interpretation. The global reference system, defined at floor level, followed the standard convention used in the SMART-DX system, with the Y-axis oriented upwards, the X-axis directed forward, and the Z-axis pointing to the right.

**Figure 1:**
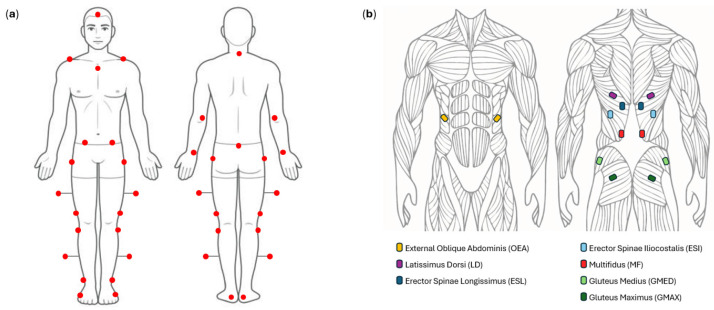
Panel (**a**): Anterior and posterior views of the 28 reflective markers (red dots) positioned on anatomical landmarks for motion capture are shown. Twenty-two markers were placed according to the Davis protocol [19], and six additional markers were placed on the sternum, head, and upper limbs. Panel (**b**): Surface EMG electrodes were placed bilaterally according to the SENIAM guidelines [20] on the following seven muscles: latissimus dorsi (LD), erector spinae iliocostalis (ESI), erector spinae longissimus (ESL), external oblique abdominis (OEA), multifidus (MF), gluteus medius (GMED), and gluteus maximus (GMAX).

During the test, we recorded, at a sampling frequency of 100 Hz, the position of 28 markers (Figure 1). Twenty-two markers were placed according to the Davis protocol [19] on the sacral spinal process, the C7 spinal process, bilaterally on the acromion, anterior superior iliac spine (ASIS), greater trochanter, lateral femoral epicondyle, fibular head, lateral malleolus, fifth metatarsal joint on the lateral foot surface, and the heel. Four markers were placed bilaterally using 5 cm bars positioned halfway between the greater trochanter and lateral femoral epicondyle and between the fibular head and lateral malleolus. Six additional markers were placed on the sternum, head, bilaterally on the lateral epicondyle of the elbow, and on the ulnar styloid process. The sEMG activity was recorded bilaterally and simultaneously from seven muscles: latissimus dorsi (LD), erector spinae iliocostalis (ESI), erector spinae longissimus (ESL), external oblique abdominis (OEA), multifidus (MF), gluteus medius (GMED), and gluteus maximus (GMAX), corresponding to fourteen EMG channels in total (Figure 1). Bipolar surface electrodes were used, and signals were recorded using differential amplification, with each channel referenced locally within the electrode pair. The muscles were recorded with a sampling frequency of 1 kHz using the BTS POCKETEMG system (BTS Bioengineering, Milan, Italy). The surface electrodes were placed following the recommendations of SENIAM guidelines [20]. Both the sEMG and kinematic data were hardware synchronized through the BTS POCKETEMG system, enabling simultaneous recording of the two signals.

### 2.4. Data Analysis

To characterize STS performance, we analyzed two complementary sets of variables: kinematic metrics and muscle activation profiles. Kinematic analysis focused on joint angles in the sagittal plane (hip, knee, and ankle flexion-extension) and trunk angles in both the frontal and sagittal plane, computed bilaterally to assess between-side asymmetries throughout the movement cycle. Movement duration was also computed for each repetition to quantify task speed. Muscle activation of the seven selected muscles was characterized through normalized EMG envelopes, analyzed bilaterally to identify side-dependent differences in activation amplitude and temporal modulation across the STS cycle.

#### 2.4.1. Kinematic Signals Analysis

The markers’ trajectories were smoothed with a fourth-order Butterworth filter with a 20 Hz cutoff frequency. Filtered marker coordinates were used to segment the movement and compute joint angles.

*Movement segmentation.* To segment the movement and identify specific events of each STS repetition, we used the speed of the markers placed on C7 and on the greater trochanter. Each repetition included both the sit-to-stand and the subsequent return-to-sit phase, and the segmentation was defined over the full movement cycle. Specifically:

- Start, as the time instant at which the anteroposterior (X) component of C7 speed reaches 10% of its peak value.
- Lift-off, as the time instant at which the markers on the greater trochanter reached 10% of their total vertical (Y) displacement.
- Stand, as the time instant at which the C7 marker reaches its maximum vertical (Y) position.
- End, as the time instant at which the anteroposterior (X) component of C7 speed fell below 10% of its peak value.

The sit-to-stand phase was defined as the interval between Start and Stand, while the return-to-sit phase is the interval from Stand to End. To quantify the time required to complete the STS, we computed the movement duration as the time interval between the Start and End events identified during movement segmentation, corresponding to the full execution of the sit-to-stand and return-to-sit cycle.

*Joint angles.* The STS is a movement that occurs predominantly in the sagittal plane; thus, we computed the flexion-extension of the trunk and hip, knee, and ankle joint angles. Additionally, trunk side bending (frontal-plane lateral flexion angle) was evaluated to assess potential asymmetries toward one side of the body.

Joint angles in the sagittal plane were calculated in OpenSim (version 4.5) using the Rajagopal model, a full-body musculoskeletal model with muscle-actuated lower extremity and torque-actuated torso/upper extremity for use in dynamic simulations of human movement [21]. The model was modified to consider the markers acquired through the stereo-photogrammetric system, allowing us to derive the following joint angles: knee and hip flexion-extension, and ankle dorsi-plantar flexion. The trunk vector was projected onto the sagittal (X–Y) and frontal (Y–Z) planes of the global coordinate system. Trunk angles were defined as the orientation of the projected vector with respect to the vertical (Y) axis and computed using a two-argument arctangent function. Specifically, sagittal-plane trunk angle was calculated as

θsag =atan2(X,Y),(1)

using the anteroposterior (X) and vertical (Y) components, whereas frontal-plane trunk angle was calculated as

θfront =atan2(Z,Y),(2)

using the mediolateral (Z) and vertical (Y) components. Positive sagittal-plane values indicated forward trunk flexion, whereas positive frontal-plane values indicated trunk lateral flexion toward the positive Z direction of the global reference system.

All the angles were then segmented, considering windows starting with the Start of the movement and finishing in correspondence with the End of the movement.

#### 2.4.2. Muscle Signals Analysis

Prior to envelope extraction, raw EMG traces were visually inspected and transient high-amplitude spikes were attenuated using a clipping procedure [22]. Specifically, samples exceeding a predefined amplitude threshold were locally replaced within a short window using the channel median value while preserving signal polarity. EMG signals were then band-pass filtered using a FIR filter (40–450 Hz), rectified, and low-pass filtered with a second-order Butterworth filter (cutoff frequency 4 Hz) to obtain the EMG envelope [23]. The envelopes were then segmented according to kinematics. For the analysis of both kinematic and sEMG data, we then considered a window starting 500 ms prior to the start and ending 500 ms after the end of the movement trial. To allow comparison across movements of different durations, kinematic and sEMG data were time-normalized by resampling each signal to 101 equally spaced points [24] using a nearest neighbor approach. The EMG envelopes were segmented according to the kinematic phases described above. To compare the modulation in amplitude of different participants and the two sides of the body of the same participant, we normalized the envelopes for their maximum value recorded over all the data collected from each participant [25]. Therefore, values represent relative EMG amplitude, with 1 corresponding to the highest activity observed for that muscle. All comparisons thus reflect relative amplitude and temporal modulation of muscle activity, rather than absolute activation levels.

Given the challenges associated with obtaining consistent maximal activations due to the clinical characteristics of the post-stroke population and the functional nature of the task, normalization to the maximum value recorded during the experimental session was adopted instead of maximal voluntary contractions (MVCs). This approach enables consistent comparison of temporal modulation patterns within each subject and between body sides; however, due to electrode-specific factors, comparisons should be interpreted in terms of relative activation profiles rather than absolute EMG amplitude. Alternative normalization strategies (e.g., normalization to the median activation across trials or to baseline activity) were also tested and did not substantially alter the observed temporal patterns.

#### 2.4.3. Statistical Analysis

Statistical analyses were designed to evaluate differences between the more impaired and less impaired sides of the body. Since the primary aim of the study was to investigate side-dependent neuromuscular modulation, all statistical tests focused on comparing the two sides within participants. The sit-to-stand task was performed under two arm conditions (free arms and crossed arms) to observe whether the side-dependent patterns were consistent under different task constraints. However, the study was not intended to test differences between arm conditions. Given the limited sample size, conducting additional statistical comparisons between conditions would have increased model complexity and the risk of Type I error. Therefore, statistical analyses were restricted to between-side comparisons.

To investigate joint angles and muscle activity as continuous functions of the movement cycle, we applied the Statistical Parametric Mapping (SPM) approach [26], which is widely used to assess statistical differences across entire time-normalized curves [27]. All analyses were conducted using the SPM1D open-source MATLAB (version 2023b) toolbox (spm1d.org). For each participant and condition, repeated sit-to-stand trials were averaged prior to statistical testing to obtain a single representative time-normalized curve for each side of the body. In one participant, EMG signals were not usable in one of the task conditions due to technical issues during acquisition. This participant was therefore excluded from the corresponding EMG analysis for that specific condition only, resulting in a different number of participants across conditions (*n* = 7 vs. *n* = 6).

Given the small sample size, between-side comparisons were performed using non-parametric, permutation-based paired *t*-tests. Permutation testing does not rely on specific distributional assumptions and generates the reference distribution empirically through repeated resampling of the observed data, making it particularly suitable for small samples and exploratory studies. All possible permutations were evaluated for each analysis; therefore, the number of unique permutations depended on the number of participants included in the specific comparison. The paired design accounted for within-subject comparisons, thereby controlling for inter-subject variability.

The significance level (α), representing the probability of incorrectly rejecting the null hypothesis (Type I error), was set at 0.05. To account for multiple comparisons across variables within each condition, a false discovery rate (FDR) correction was applied using the Benjamini–Hochberg procedure. In the Results section, cluster-level *p*-values from SPM analyses are reported prior to FDR correction, and the effects that remain significant after correction within each condition are explicitly indicated.

## 3. Results

### 3.1. Kinematic Performance

All participants were able to complete the task in the free arm condition and six out of seven in the crossed arm condition. Indeed, ID3, the subject with the greatest difficulty in performing the task, was able to complete it only maintaining free hands.

**Figure 2:**
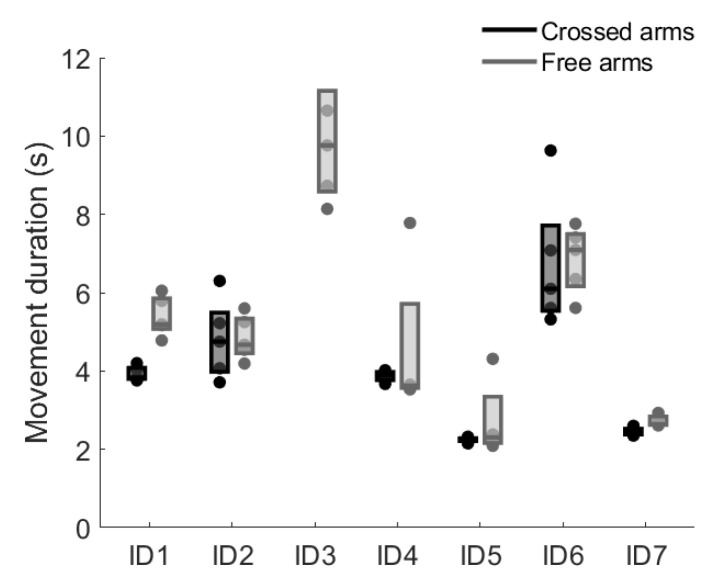
Movement durations of the STS task with crossed arms (black) and free arms (gray) for each subject. Each dot represents a single repetition, while box plots show the interquartile range and the median for each condition.

For participants ID1, ID2, ID4, ID5, and ID7, movement durations ranged between 2 and 8 s, while ID3 and ID6 showed prolonged durations, reaching up to 12 s (Figure 2).

STS times were relatively consistent between the two conditions for ID2, ID6, and ID7, whereas ID4 and ID5 exhibited greater variability in the free arms condition.

**Figure 3:**
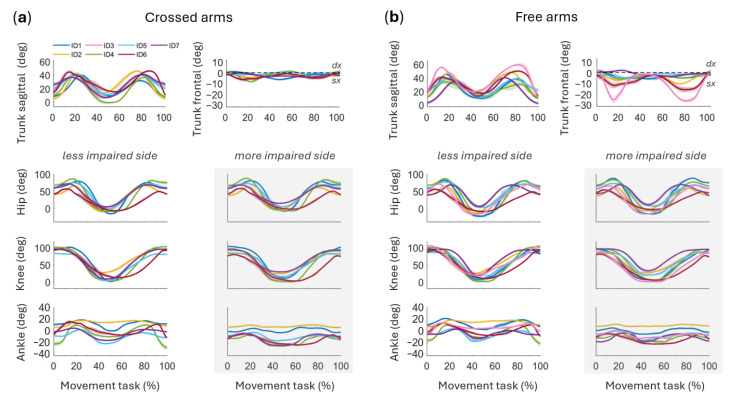
Panels (**a**,**b**) show individual joint kinematics (mean ± standard error for each participant) across the normalized STS cycle under crossed arms (**a**) and free arms (**b**) conditions. The first row displays trunk angles in the sagittal (**left**) and frontal (**right**) planes, followed by hip, knee, and ankle sagittal-plane angles. Data are presented separately for the less impaired side (white background) and the more impaired side (gray background).

Kinematic analysis revealed a consistent pattern across all participants in both conditions independently of the arm positions, with distinct phases throughout the STS task (Figure 3). The preparatory phase, lasting from task initiation to approximately 20% of the STS cycle, is characterized by slight hip flexion (10–30°), ankle dorsiflexion (5–15°), and trunk flexion in the sagittal plane (20–45°). This coordinated motion prepares the body for the subsequent transition. The rising phase (20–50% of STS cycle) begins as the subject lifts off the chair and involves trunk extension (15–35°), hip extension (70–90°), knee extension (60–80°), and ankle plantarflexion (15–20°) (Figure 3).

These movements reach a peak when the subject achieves a fully upright posture, typically at around 50% of the movement cycle. The lowering phase (50–80% of STS cycle), returning to the seated position, consists of trunk flexion, hip flexion, knee flexion, and ankle dorsiflexion, concluding when the subject contacts the chair at approximately 80% of the task. The final phase (80–100% of STS cycle), a transition back to the starting position, includes trunk extension, hip extension, and ankle plantarflexion (Figure 3).

In the sagittal plane, trunk angles show little variation across participants, except for ID3, who shows marked trunk flexion during the STS task with free arms (Figure 3b), and ID4, who shows increased trunk extension in the crossed arms condition (Figure 3a). In the frontal plane, trunk angles stay close to zero, exhibiting minor oscillations (<10°) during both the rising and sitting phases in the crossed arms condition (Figure 3a). However, in the free arms condition, larger variations occur, particularly for ID2, ID3, and ID6, with lateral shifts toward the less impaired side (Figure 3b). Hip and knee angles exhibit consistent patterns across participants with minor variations. ID6 shows reduced hip and knee range of motion during both the rising and sitting phases, as reflected by the flatter trajectories of the joint angles compared to the other participants (Figure 3).

ID1, ID5, and ID7 exhibit slightly greater extension of the less impaired side during the standing phase. ID3 and ID6 show a knee angle offset because their less impaired leg was held back relative to the more impaired leg, resulting in a greater left knee angle at the start of the movement (Figure 3). Ankle angles show side-to-side asymmetry, with the left foot exhibiting greater dorsiflexion in all participants except ID5 (Figure 3a). This trend persists in the free arms condition (Figure 3b). Additionally, ankle angles often show an offset, with the less impaired foot positioned slightly behind the more impaired foot, leading to higher angles on the less impaired side (Figure 3).

**Figure 4:**
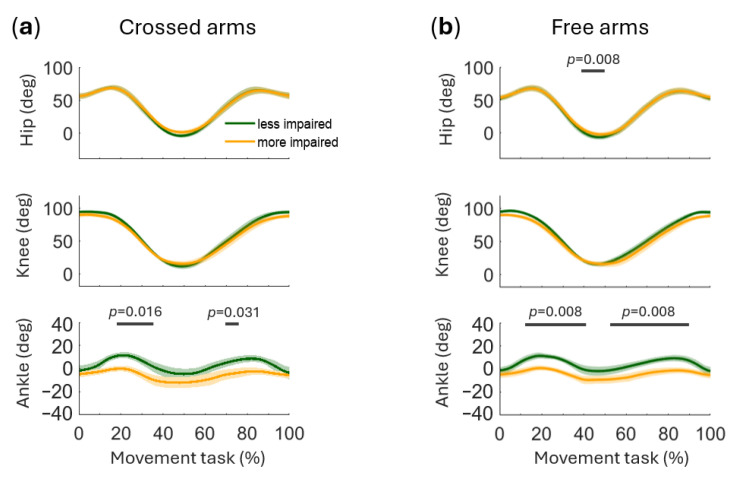
Panels (**a**,**b**) present group-level comparisons between sides (mean ± standard error across participants) for the crossed arms (**a**) and free arms (**b**) conditions. Statistical parametric mapping (SPM) results are reported as horizontal lines over the significant time intervals. The *p*-value for each interval is reported near the corresponding band. In these panels, the less impaired side is shown in dark green and the more impaired side in light orange.

Hip, knee, and ankle joint angles in the sagittal plane were compared between the more and less impaired sides under the crossed arms and free arms conditions (Figure 4). Statistical analysis revealed a significant difference in hip angle around 40–50% of the movement cycle during the free arms condition (*p* = 0.008) (Figure 4b), with no corresponding effect in the crossed arms condition (*p* = 0.094).

No significant differences were observed in knee angles in either condition (free arms: *p* = 0.125, crossed arms: *p* = 0.188) (Figure 4).

In the crossed arms condition, ankle angles differed significantly at approximately 30% (*p* = 0.016) and 70% (*p* = 0.031) of the movement cycle (Figure 4a). In the free arms condition, significant differences occurred at the same phases but persisted over a longer portion of the cycle (*p* = 0.008) (Figure 4b). After applying FDR correction for multiple comparisons, all significant differences identified by SPM remained significant.

### 3.2. Muscle Activity

**Figure 5:**
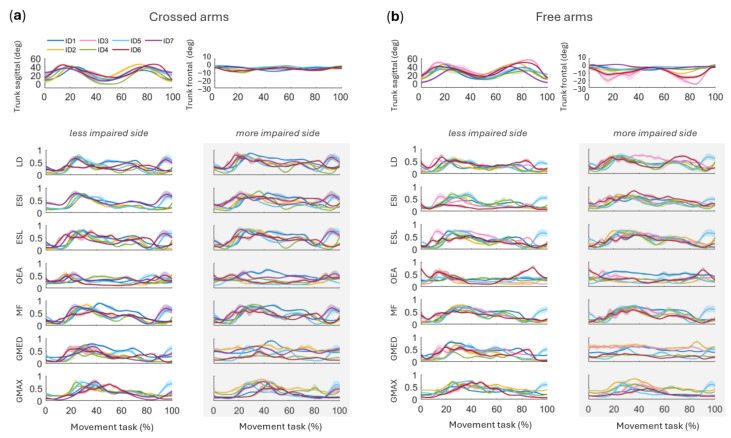
Trunk and gluteal muscle activation during the STS task. Panels (**a**,**b**) show individual muscle activation profiles (mean ± standard error for each participant) across the normalized STS cycle under crossed arms (**a**) and free arms (**b**) conditions. Data are presented separately for the less impaired side (white background) and the more impaired side (gray background). Sagittal- and frontal-plane trunk angles (first row) are included as a kinematic reference for movement phase identification. Abbreviations: LD, latissimus dorsi; ESI, erector spinae iliocostalis; ESL, erector spinae longissimus; OEA, external oblique abdominis; MF, multifidus; GMED, gluteus medius; GMAX, gluteus maximus.

Due to acquisition problems, EMG data were missing for participant ID7 in the free arms condition; this dataset was excluded from the corresponding analyses. Regardless of condition, LD, ESI, ESL, and MF muscles showed similar temporal patterns (Figure 5), with activity mainly occurring between 20% and 80% of the movement cycle. A major peak was consistently observed around 30%, followed by a smaller peak around 70%. OEA displayed a flatter profile, with two smaller peaks at approximately 15% and 85% of the cycle. GMED was most active between 20% and 80%, peaking around 30%, whereas GMAX exhibited a pronounced peak at approximately 40% of the cycle.

**Figure 6:**
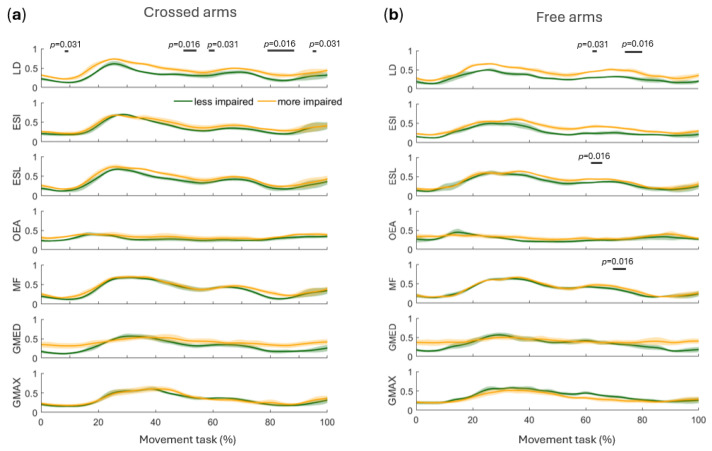
Panels (**a**,**b**) present group-level comparisons between sides (mean ± standard error across participants) for the crossed arms (**a**) and free arms (**b**) conditions. The less impaired side is shown in dark green and the more impaired side in light orange. Statistical parametric mapping (SPM) results are reported as horizontal lines over significant time intervals. The *p*-value for each interval is reported near the corresponding band. Abbreviations: LD, latissimus dorsi; ESI, erector spinae iliocostalis; ESL, erector spinae longissimus; OEA, external oblique abdominis; MF, multifidus; GMED, gluteus medius; GMAX, gluteus maximus.

A qualitative comparison of the activation profiles across the two task conditions revealed largely comparable temporal patterns of muscle activity (Figure 5). Although the crossed arms condition tended to show slightly higher activation levels with more distinct peaks (Figure 5a), these differences were modest and were not formally tested statistically, as the study was not designed to evaluate condition-related effects. Consistent with this observation, SPM analyses detected fewer significant side-to-side differences in the crossed arms condition (Figure 6). Specifically, only LD differed significantly around 10% (*p* = 0.031), 50% (*p* = 0.016) and 80% (*p* = 0.016) of the movement cycle (Figure 6a). No significant side-to-side differences were observed for the other muscles, including ESI (*p* = 0.312), ESL (*p* = 0.094), OEA (*p* = 0.438), MF (*p* = 0.094), GMED (*p* = 0.094), and GMAX (*p* = 0.688). This suggests that side-dependent differences can be described without explicitly stratifying the results by condition, given the largely comparable activation patterns between crossed and free arms trials.

*Side-dependent modulation.* Despite inter-individual variability, LD showed a consistent side-dependent modulation, with a greater relative contribution on the more impaired side during both the rising (20–50% of the cycle) and lowering phases (50–80%) (Figure 5). At the group level, SPM confirmed significant side-to-side differences for LD in the free arms condition around 60% (*p* = 0.031) and 80% of the cycle (*p* = 0.016) (Figure 6b). In the crossed arms condition, LD differed around 10% (*p* = 0.031), 50% (*p* = 0.016) and 80% (*p* = 0.016) of the cycle (Figure 6a). At the individual level, this modulation was evident across most participants.

A similar side-dependent trend was observed for ESI in ID1, ID2, ID3, ID5, and ID6, and for ESL in ID1, ID2, ID4, ID5, and ID7 (Figure 5). Importantly, only ESL showed significant group-level differences in the free arms condition (60% of the cycle, *p* = 0.016), whereas ESI did not reach significance (*p* = 0.094) (Figure 6b).

In ID3, LD, ESI, and ESL showed earlier activation on the less impaired side, with a peak around 15% of the cycle that was absent on the more impaired side. The modulation of OEA was variable across participants: it was more pronounced on the more impaired side in ID1 but showed an early peak (~15% of the cycle) on the less impaired side in ID2, ID3, and ID4 (Figure 5). At the group level, however, OEA did not show significant side-to-side differences (*p* = 0.625) (Figure 6).

MF activation patterns were generally comparable between sides, except in ID4, ID6, and ID7, where the more impaired side showed a relatively greater contribution during the late phases of the cycle (Figure 5). This effect was supported by SPM, where MF showed significant side-to-side differences around 70% of the cycle in the free arms condition (*p* = 0.016) (Figure 6b). GMED exhibited reduced temporal modulation on the more impaired side, resulting in a flatter activation profile across the STS cycle for ID1, ID2, ID3, ID4, ID6, and ID7 (Figure 5); however, this effect was not statistically significant at the group level (*p* = 0.219) (Figure 6).

GMAX on the more impaired side often exhibited a shorter activation duration, with a relative decline in activity after 40–60% of the STS cycle in ID1, ID3, ID5, and ID6. Participant ID4 showed comparable temporal profiles between sides, whereas ID2 exhibited a more pronounced relative contribution around 40% of the cycle on the more impaired side (Figure 5). At the group level, GMAX did not show significant side-to-side differences (*p* = 0.438) (Figure 6). After FDR correction for multiple comparisons, all significant side-to-side differences observed in the free arms condition were confirmed. In the crossed arms condition, the difference observed for LD was no longer significant after correction.

## 4. Discussion

This study investigated STS performance in individuals with right-sided hemiparesis by leveraging a synchronized, multimodal sensing setup that combines optoelectronic motion capture and multichannel surface electromyography. This integrated acquisition enabled a time-resolved characterization of joint/segment kinematics together with trunk and gluteal muscle activation profiles across repeated STS cycles. To our knowledge, this is among the first studies to provide a synchronized, phase-resolved sensor-based characterization of trunk muscle modulation during STS in individuals with chronic hemiparetic stroke.

Trunk control is widely recognized as a key component of postural stability and asymmetry management after stroke [12], yet it has received comparatively limited attention in STS analyses [15]. In this context, we extend prior STS analyses by jointly quantifying phase-dependent kinematic asymmetries and side-dependent trunk muscle modulation across repeated STS cycles. Results revealed that post-stroke STS is characterized by a progressive proximal shift in compensatory involvement across the movement cycle, with trunk muscles playing a particularly prominent role during the phases that most challenge postural stability under asymmetric loading.

From a temporal perspective, results revealed considerable variability across subjects in movement duration (Figure 2), with values exceeding those typically reported in unimpaired adults [4,9]. This probably reflects a strategy to reduce the mechanical demands of the task by decreasing movement velocity, thereby allowing more time for postural adjustments and weight transfer under conditions of reduced neuromuscular capacity [28]. The high inter-subject variability in movement duration further suggests that individuals adopt different temporal strategies depending on their residual motor function, consistent with the heterogeneity of compensatory patterns observed across participants. Asymmetries in both kinematics and muscle activity emerged throughout the movement cycle, supporting the view that post-stroke STS is not characterized by a single compensatory pattern but rather by phase-specific strategies [6]. During the initial rising phase, around 30% of the cycle, the ankle on the less impaired side showed greater dorsiflexion (Figure 4), likely to facilitate seat-off and provide a more stable support base. This asymmetric ankle angle may reflect an active strategy to shift the center of mass anteriorly and increase reliance on the less impaired limb prior to seat-off, redistributing the mechanical demands toward that side of the body. At the knee, a tendency toward greater extension on the less impaired side was observed in some participants (Figure 3), although this did not reach statistical significance (Figure 4). Previous work focusing on distal lower-limb muscles has documented early inter-limb alterations at movement initiation, notably delayed tibialis anterior activation and premature/excessive soleus activity in the affected limb [8]. Lee et al. [1] further demonstrated that during STS in chronic stroke survivors, muscles on the unaffected side exhibit faster reaction times than those on the affected side, with delayed activation of deep abdominal muscles, specifically the transverse abdominis and internal oblique, relative to leg muscles on the affected side, suggesting a broader reorganization of neuromuscular control that extends beyond the distal lower limb.

By contrast, in the present cohort, we did not observe consistent side-dependent modulation in the recorded proximal muscles (gluteal and trunk) during the early rising phase (Figure 6). This may indicate that deficits in this phase are expressed more prominently at the distal level or are affected by the inter-subject heterogeneity and the low sample size of the population. This dissociation between distal and proximal neuromuscular asymmetries in the early rising phase may reflect a hierarchical organization of compensatory recruitment, whereby distal adjustments are prioritized to ensure successful seat-off, while proximal trunk stabilization becomes more critical in later phases.

At the point of reaching the standing position, around 50% of the cycle, kinematic asymmetries shifted proximally to the hip, which was significantly more extended on the less impaired side (Figure 4b). This is consistent with the progressive transfer of gravitational load toward the standing configuration: as the body approaches full extension, the demands on hip extensors increase, and the reduced capacity of the more impaired gluteal musculature forces greater reliance on the less impaired limb for vertical propulsion [29]. At this stage, the gluteus maximus showed side-dependent modulation patterns (Figure 5), although these differences were not statistically significant (Figure 6). This is in line with Nam et al. [16], who reported greater gluteus maximus activation on the affected side during the standing phase of STS when the affected foot was placed posteriorly, a foot positioning strategy spontaneously adopted by some participants in our cohort as well. This progressive proximal involvement extended further to the trunk level: Nam et al. [16] also reported greater erector spinae activation on the affected side in the same condition. Although we did not observe significant side-dependent differences in erector spinae at this phase of the movement, latissimus dorsi showed a greater relative contribution on the more impaired side (Figure 6).

The recruitment of latissimus dorsi on the more impaired side at this stage may reflect an anticipatory postural adjustment, whereby the nervous system pre-activates trunk stabilizers to prepare for the increased postural demands of the subsequent lowering phase. This interpretation is consistent with the known role of latissimus dorsi as a global trunk stabilizer contributing to controlled movement [17]. Taken together, these findings suggest an early recruitment of trunk musculature as part of a compensatory stabilization strategy at the point of reaching the standing position. The return to the sitting phase, around 50–80% of the cycle, showed the most pronounced compensatory strategies. Distally, ankle differences re-emerged, with greater dorsiflexion on the less impaired side, indicative of more effective eccentric control (Figure 4). The observed asymmetry, already identified during the rising phase, further confirms a disproportionate load distribution between the two sides of the body also during the lowering phase, with the more impaired limb contributing less effectively to the deceleration of the body toward the seat.

This finding is consistent with previous evidence of significant bilateral asymmetries in ankle joint angle during the STS task in individuals with stroke during both the rising and sitting phases [30]. Proximally, trunk muscles, particularly latissimus dorsi, erector spinae, and multifidus, exhibited significant side-dependent modulation, characterized by a prolonged relative contribution on the more impaired side (Figure 6). This finding suggests that, when eccentric lower-limb control is reduced on the more impaired side, trunk musculature is recruited to compensate by providing additional postural stabilization through increased activation of the posterior trunk muscles. Notably, the proximal compensatory pattern was not observed during the rising phase (Figure 6). The difference likely reflects the distinct biomechanical demands of the task: the rising phase is propulsion-driven and requires coordinated concentric force generation from both lower limbs, thereby limiting compensatory strategies, whereas the lowering phase relies on controlled eccentric modulation, allowing greater flexibility in coordination across body segments and facilitating increased recruitment of trunk musculature when lower-limb control is reduced. This interpretation aligns with reports of increased weight distribution asymmetry and reduced stabilization during the lowering phase of STS after stroke [6] and with evidence of stroke-related alterations in trunk muscle activation during STS [1,16] as well as during other trunk motor tasks [17,18].

The reduced and shortened gluteus maximus activation on the more impaired side observed in several participants (Figure 5) complements evidence from Cheng et al. [8] and Boukadida et al. [9], who identified delayed and reduced paretic muscle recruitment as primary determinants of STS performance after stroke. The flatter gluteus medius activation profile on the more impaired side may reflect deficits in frontal-plane stabilization, consistent with the increased frontal-plane trunk excursions observed in some participants. Although these lower-limb differences did not reach group-level significance (Figure 6), likely due to inter-subject heterogeneity and small sample size, they reinforce the picture of a predominantly less-impaired-side-driven STS strategy, with the more impaired side contributing less effectively to both propulsion and stabilization.

Analysis at the individual level further highlighted the heterogeneity of movement strategies. Participants such as ID5 and ID7 demonstrated shorter STS durations (Figure 2) and fewer compensatory features, with asymmetries largely confined to distal joint kinematics (Figure 3). In contrast, participants ID3 and ID6 required substantially longer movement durations (Figure 2) and exhibited greater trunk excursions in the frontal plane (Figure 3), together with altered modulation of both gluteal and trunk muscles (Figure 5). These subject-specific behaviors help explain why some biomechanical variables did not show consistent group-level differences, as individual compensatory strategies varied across participants. At the same time, the group-level SPM analysis revealed recurring side-dependent modulation patterns across participants, particularly in trunk muscles such as the latissimus dorsi, erector spinae longissimus, and multifidus. Considering both individual activation profiles and group-level trends therefore helped contextualize the results by distinguishing subject-specific adaptations from neuromuscular patterns that were more consistently observed across participants despite the limited sample size.

When qualitatively comparing free arms and crossed arms conditions, the free arms condition accentuated lateral body shifts toward the less impaired side, particularly in the trunk angle in the frontal plane. In this condition, side-specific differences in kinematics and muscle activity were more pronounced, suggesting that allowing upper-limb freedom may reveal compensatory strategies. In contrast, the crossed arms condition showed slightly higher activation levels with clearer peaks across all muscles and in both sides of the body, suggesting increased postural effort due to restricted upper-limb assistance.

*Clinical applications and future perspective.* The findings of this study provide new insights into post-stroke movement analysis, particularly by incorporating a comprehensive evaluation of trunk muscle activations, an aspect that has been less explored in the previous literature. While studies have focused primarily on the lower limb during the STS task [1,8,9,10,11], our results emphasize the critical role of trunk control and its asymmetric activation patterns in post-stroke individuals. The significant differences observed between sides of the body in trunk muscle activation patterns suggest that postural adjustments and compensatory strategies involve not only the lower limbs but also the upper body, contributing to movement inefficiencies and asymmetries.

From a clinical perspective, these findings may contribute to a more comprehensive evaluation of functional performance after stroke. The integration of kinematic and EMG sensing allows clinicians to identify asymmetries not only in joint motion but also in neuromuscular activation patterns, potentially revealing compensatory strategies that are not easily detectable through observational assessment alone. In particular, the identification of phase-specific trunk muscle modulation may help clinicians better understand how stroke survivors redistribute mechanical demands during the different phases of the STS movement. In addition, the sensor-derived metrics used in this study could support the development of instrumented assessment tools for rehabilitation. Quantitative indicators derived from synchronized motion capture and EMG signals may enable objective monitoring of progress over time and provide measurable markers of motor recovery or compensatory behavior. Such approaches could facilitate the evaluation of rehabilitation interventions aimed at improving movement symmetry, trunk control, and overall functional efficiency during everyday tasks.

Since sEMG can also provide information on muscle fatigue, future studies could investigate fatigue-related changes during repeated sit-to-stand tasks. Several EMG-based approaches have been proposed to estimate muscle fatigue during dynamic movements, including methods based on spectral or amplitude changes in the signal over time [31]. Although the present study focused on activation patterns across the movement cycle, incorporating fatigue-related metrics in future work may provide further insight into how neuromuscular strategies evolve during repeated functional movements in post-stroke individuals.

This study has some limitations. The sample size was inherently limited by the study design. As an exploratory investigation, the study was designed to generate hypotheses rather than provide confirmatory evidence, thereby limiting statistical power and generalizability. Accordingly, the analysis focused on identifying phase-specific, sensor-derived trends rather than establishing definitive causal relationships. In such a small cohort, inter-individual variability may strongly influence group-level trends, making it difficult to clearly distinguish between consistent neuromuscular patterns and subject-specific compensatory behaviors. The cohort was also not gender-balanced, which further limits the extension of these observations to the broader post-stroke population. EMG signals were normalized to the maximum activation observed during the experimental session rather than to maximal voluntary contractions. While this approach was adopted because reliable MVC measurements may be difficult to obtain in post-stroke individuals, it may limit comparisons of absolute activation levels across participants and between sides. Additionally, ground reaction forces were not recorded, which would have allowed direct quantification of loading asymmetries. Future studies integrating kinetic measurements, and larger and more balanced cohorts will be necessary to validate and extend these preliminary observations.

Future research should further investigate how different rehabilitation strategies influence trunk control and overall movement efficiency, potentially leading to more targeted interventions aimed at reducing compensatory behaviors and improving functional outcomes in post-stroke individuals.

## Data Availability

The data presented in this study is available on reasonable request from the corresponding author.

## Abbreviations

The following abbreviations are used in this manuscript:

LD	Latissimus Dorsi
ESI	Erector Spine Iliocostalis
ESL	Erector Spine Longissimus
OEA	Oblique External Abdominis
MF	Multifidus
GMED	Gluteus Medius
GMAX	Gluteus Maximus
